# Bumetanide treatment during early development rescues maternal separation-induced susceptibility to stress

**DOI:** 10.1038/s41598-017-12183-z

**Published:** 2017-09-19

**Authors:** Die Hu, Zhou-Long Yu, Yan Zhang, Ying Han, Wen Zhang, Lin Lu, Jie Shi

**Affiliations:** 1 National Institute on Drug Dependence and Beijing Key Laboratory of Drug Dependence, Peking University, Beijing, 100191 China; 2 Department of Pharmacology, School of Basic Medical Sciences, Peking University Health Science Center, Beijing, 100191 China; 3 Institute of Mental Health, National Clinical Research Center for Mental Disorders, Key Laboratory of Mental Health and Peking University Sixth Hospital, Peking University, Beijing, 100191 China; 4 Peking-Tsinghua Center for Life Sciences and PKU-IDG/McGovern Institute for Brain Research, Beijing, 100191 China; 5 State Key Laboratory of Natural and Biomimetic Drugs, Beijing, 100191 China; 6 Key Laboratory for Neuroscience of the Ministry of Education and Ministry of Public Healthy, Beijing, 100191 China

## Abstract

Stress is a major risk factor for psychiatric disorders, such as depression, posttraumatic stress disorder, and schizophrenia. Early life stress, such as maternal separation, can have long-term effects on the development of the central nervous system and pathogenesis of psychiatric disorders. In the present study, we found that maternal separation increased the susceptibility to stress in adolescent rats, increased the expression of Na^+^/K^+^/2Cl^−^ cotransporter 1 (NKCC1) on postnatal day 14, and increased the expression of K^+^/2Cl^−^ cotransporter 2 (KCC2) and γ-aminobutyric acid A (GABA_A_) receptor subunits on postnatal day 40 in the hippocampus. NKCC1 inhibition by the U.S. Food and Drug Administration-approved drug bumetanide during the first two postnatal weeks rescued the depressive- and anxiety-like behavior that was induced by maternal separation and decreased the expression of NKCC1, KCC2 and GABA_A_ receptor α1 and β2,3 subunits in the hippocampus. Bumetanide treatment during early development did not adversely affect body weight or normal behaviors in naive rats, or affect serum osmolality in adult rats. These results suggest that bumetanide treatment during early development may prevent the maternal separation-induced susceptibility to stress and impairments in GABAergic transmission in the hippocampus.

## Introduction

Stress refers to the effects of internal and external environmental factors that seriously threaten homeostasis^1^. Stressors have a major influence on mood, one’s sense of well-being, behavior, and health^2^. Individual responses to stress vary widely. Some individuals develop trauma-related mental illnesses, such as posttraumatic stress disorder and depression. Others develop mild to moderate psychological symptoms, whereas others do not present negative emotions^3^. Numerous studies suggest that the interaction between genetic factors and early-life environmental factors can affect individual epigenetic modifications, gene expression patterns, and brain development, which may lead to higher susceptibility to mental illness when exposed to environmental stress during adolescence or adulthood^4–6^. Therefore, it is considered that neuropsychiatric diseases are results of two (or more) stressors experienced over the lifespan, as stated in the Two/Multiple-Hit hypothesis^7–10^. Early life stress (ELS) is a major risk factor for mental health problems at all stages of life^11^. Previous studies have shown that ELS can lead to multiple behavioral abnormalities in adolescence and adulthood, such as depression and anxiety-like behavior^12^, learning and memory deficits^13^, and eating disorders^14^. Clinical and preclinical studies have found that ELS has a considerable influence on brain development, particularly hippocampal volume and synaptic plasticity^15–17^.

γ-Aminobutyric acid (GABA) is the major inhibitory neurotransmitter in the central nervous system in adults. However, GABA can cause chloride ion (Cl^−^) efflux and excite immature neurons during early development^18,19^. The shift from excitatory to inhibitory neurotransmission mainly depends on a family of cation-chloride cotransporters (CCCs), particularly Na^+^/K^+^/2Cl^−^ cotransporter 1 (NKCC1) and K^+^/2Cl^−^ cotransporter 2 (KCC2). In immature neurons, the intracellular Cl^−^ concentration ([Cl^−^]_i_) is mainly driven by NKCC1 while KCC2 expression is low. Therefore, neurons exhibit high [Cl^−^]_i_, causing Cl^–^ efflux and postsynaptic depolarization when GABA acts on neurons. The greater expression of functional KCC2 during neuronal maturation results in lower [Cl^−^]_i_, causing Cl^−^ influx when GABA_A_ receptors are activated^18^. In the human neocortex, KCC2 expression begins to increase 40 weeks after birth, accompanied by a decrease in NKCC1 activity and expression, resulting in the conversion of GABA signals to hyperpolarization^20^. KCC2 is widely expressed in the central nervous system, specifically in neurons, and rarely in the peripheral nervous system or non-neuronal cell types^21^. NKCC1 is expressed in both neurons and glial cells in the central nervous system^22^.

Cation-chloride cotransporters play an important role in the development of synaptic formation and neuronal plasticity^23,24^. Derangements of CCCs have been implicated in the pathogenesis of seizures and neuropathic pain^18^ and also play a key role in many psychiatric disorders, including schizophrenia, autism, Down syndrome, and Fragile X syndrome^25–28^. The NKCC1 antagonist bumetanide is a U.S. Food and Drug Administration-approved loop diuretic that has proven to be useful for the treatment of epilepsy, autism, and schizophrenia^29–31^. Bumetanide treatment during early postnatal development could reverse neonatal exposure to anesthesia (sevoflurane)-induced long-term endocrine and neurobehavioral abnormalities^32^ and also rescue a genetic epilepsy in mice^33^, however, bumetanide had poor antiepileptic efficacy in the newborn babies with hypoxic ischemic encephalopathy (NEMO) trial, which was a Phase I/II trial that assessed the safety and optimal dose of bumetanide for the treatment of acute neonatal seizures^34^. This Phase I/II failure cannot necessarily be extrapolated to other types of seizures, and bumetanide might still be a therapeutic tool to attenuate several brain disorders^35^.

Although previous clinical and animal studies reported a role for CCCs in several neurological and psychiatric disorders, still unknown is whether CCCs modulate neural maladaptation after maternal separation (MS). In the present study, we used MS as the ELS and forced swimming to simulate low levels of stress during adolescence. We investigated the effects of MS on the expression of NKCC1 and KCC2 and explored whether bumetanide reverses the susceptibility to stress that is induced by MS and alterations of the GABAergic system in adolescent and adult rats.

## Results

### Maternal separation induced depressive- and anxiety-like behavior in adolescent rats

**Figure 1:**
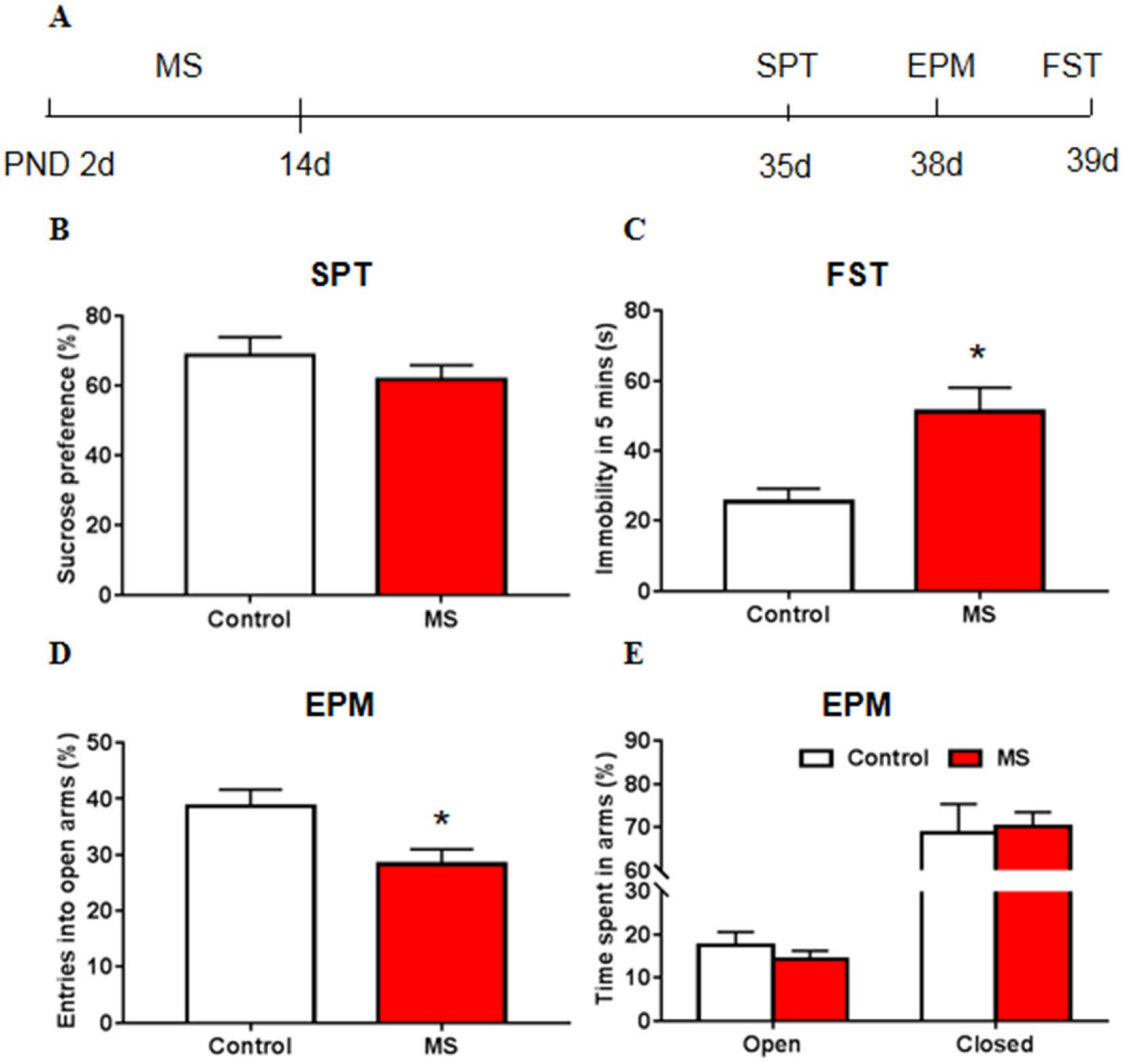
Maternal separation led to depressive- and anxiety-like behavior in adolescent rats. (**A**) Experimental timeline of maternal separation (MS) and behavioral tests. (**B**) Sucrose preference in the sucrose preference test (SPT). (**C**) Immobility time in the forced swim test (FST). (**D**) Entries into open arms in the elevated plus maze (EPM). Entries into the open arms are shown as a percentage of all entries. (**E**) Time spent in arms in the EPM. Time spent in the open or closed arms is plotted as a percentage of the total observation time; time spent in the middle is not shown. The data are expressed as mean ± SEM. n = 16–17 per group (7–8 females, 8–10 males). *p < 0.05, compared with control group.

We first used different behavioral paradigms to detect whether depressive- and anxiety-like behaviors are observed in maternally separated adolescent rats. The statistical analysis of the behavioral data was performed using Student’s *t*-test. Maternal separation had no effect on sucrose preference in the sucrose preference test (SPT; *t*_31_ = 1.068, *p* = 0.294; Fig. 1B) but significantly increased immobility time in the forced swim test (FST; *t*_28_ = 3.244, *p = *0.003; Fig. 1C). Maternal separation also led to anxiety-like behavior, reflected by significant change in the entries into the open arms (EPM; *t*_24_ = 2.516, *p* = 0.019; Fig. 1D), with no significant change in the time spent in open arms (EPM; *t*_24_ = 0.913, *p* = 0.370; Fig. 1E) or closed arms (EPM; *t*_24_ = 0.229, *p* = 0.821; Fig. 1E). The body weight of rats showed no differences between maternal separation groups and control groups at P14 and P40 (*p* > 0.05; Fig. S2). The SPT and FST are well-established behavioral models of anhedonia and despair in rodents^36,37^. However, forced swimming during adolescence can also serve as a stressor and was additionally used to test the susceptibility to stress^10^. Altogether, these results suggest that maternal separation impaired emotional behavior in adolescent rats, reflected by depressive- and anxiety-like behavior and higher susceptibility to stress.

### Maternal separation altered the expression of NKCC1, KCC2, and GABA_A_ receptor subunits in the hippocampus

**Figure 2:**
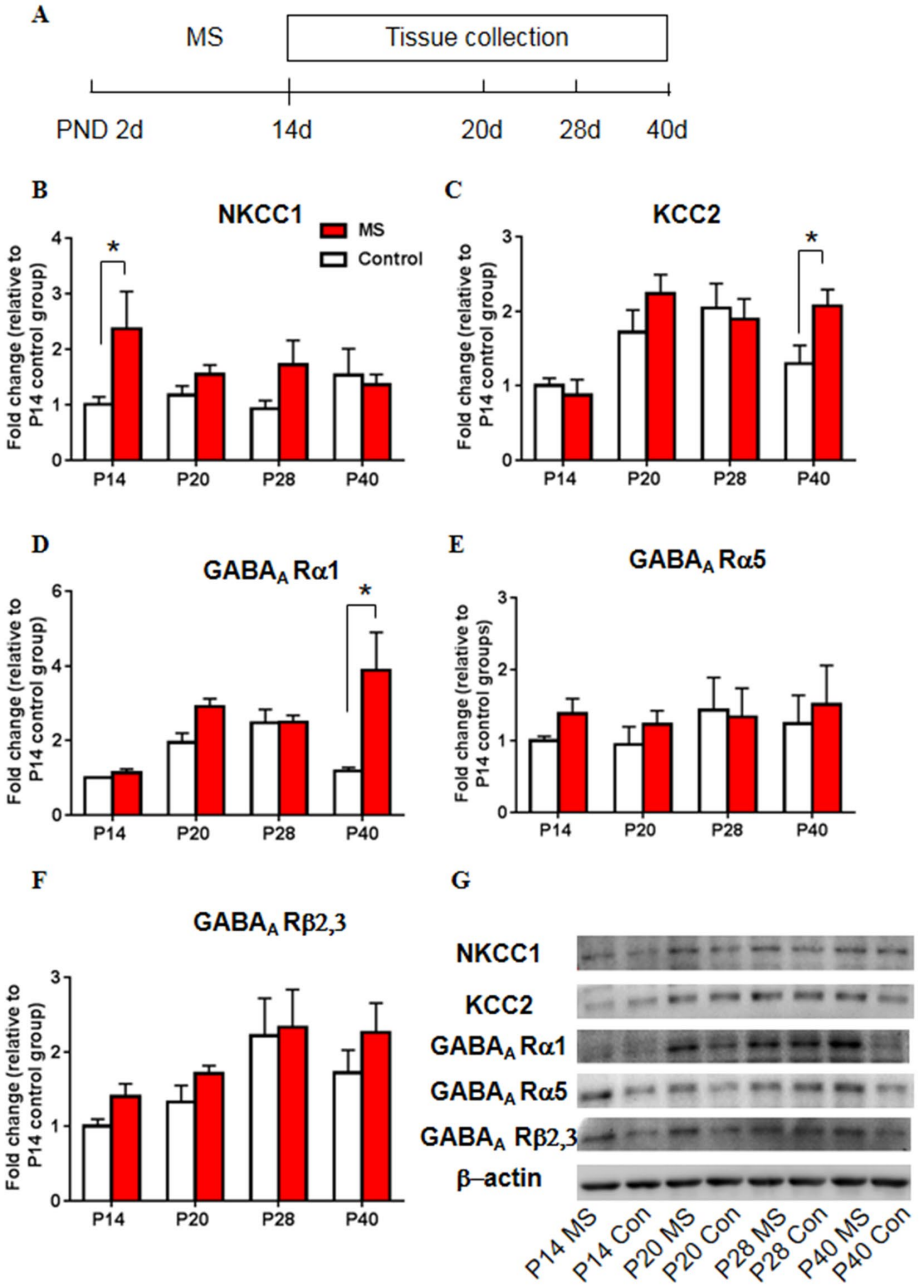
Maternal separation altered the expression of NKCC1, KCC2, and GABA_A_ receptor subunits in the hippocampus. (**A**) Experimental timeline of maternal separation and tissue collection. (**B**–**F**) NKCC1 (**B**), KCC2 (**C**), GABA_A_ receptor α1 subunit (**D**), GABA_A_ receptor α5 subunit (**E**), and GABA_A_ receptor β2,3 subunit (**F**) levels in the CA1 area of the hippocampus on postnatal days 14, 20, 28, and 40. (**G**) Representative Western blots in the CA1. Full-length blots are presented in Supplementary Figure S4. n = 10 per group (4 females, 6 males). The data are expressed as mean ± SEM. *p < 0.05, compared with control group at corresponding ages.

We next used Western blot to detect the expression of NKCC1 and KCC2 in maternally separated rats at different developmental stages (postnatal day 14 [P14], P20, P28, and P40). The two-way analysis of variance (ANOVA), with age (P14, P20, P28, P40) and stress (control and MS) as the between-subjects factors, followed by Tukey’s *post hoc* test, was used to analyze the Western blot data. A significant main effect of stress on the expression of NKCC1 was found (age: *F*_7,29_ = 0.379, *p* = 0.769; stress: *F*_7,29_ = 5.117, *p* = 0.031; age × stress: *F*_7,29_ = 1.595, *p* = 0.212; Fig. 2B). The *post hoc* test revealed that MS significantly increased the expression of NKCC1 in the CA1 of the hippocampus on P14 (*p* = 0.015; Fig. 2B), with a tendency toward an increase in NKCC1 levels at other ages. The analysis revealed a significant main effect of age on the expression of KCC2 (age: *F*_7,31_ = 6.986, *p* = 0.001; stress: *F*_7,31_ = 1.985, *p* = 0.169; age × stress: *F*_7,31_ = 1.676, *p* = 0.192; Fig. 2C). A significant difference in KCC2 expression was found between maternally separated rats and control rats on P40 (*p* = 0.036; Fig. 2C).

Maternal separation also altered the expression of several GABA_A_ receptor subunits. Significant main effects of age and stress on the expression of the GABA_A_ receptor α1 subunit were found, with a significant age × stress interaction (age: *F*_7,27_ = 4.010, *p* = 0.018; stress: *F*_7,27_ = 8.420, *p* = 0.007; age × stress: *F*_7,27_ = 3.780, *p* = 0.022; Fig. 2D). The *post hoc* test revealed that MS increased the levels of the GABA_A_ receptor α1 subunit on P40 compared with the control group (*p* < 0.001; Fig. 2D). No significant main effects or interaction on the expression of the GABA_A_ receptor α5 subunit were found (age: *F*_7,32_ = 0.766, *p* = 0.522; stress: *F*_7,32_ = 1.618, *p* = 0.213; age × stress: *F*_7,32_ = 0.403, *p* = 0.752; Fig. 2E). A main effect of age on the expression of the GABA_A_ receptor β2,3 subunit was found (age: *F*_7,31_ = 3.998, *p* = 0.016; stress: *F*_7,31_ = 2.287, *p* = 0.141; age × stress: *F*_7,31_ = 0.143, *p* = 0.933; Fig. 2F). The *post hoc* test revealed no significant differences between the MS groups and control groups, but there was a tendency toward an increase in the levels of the GABA_A_ receptor β2,3 subunit that was induced by MS. Additionally, we examined the levels of NKCC1 and KCC2 in the basolateral amygdala, but the results showed no significant changes (Fig. S1).

These results highlight the role of CCCs and GABA_A_ receptors in ELS-induced behavioral changes and identify the hippocampus as a brain area that is vulnerable to ELS.

### Systemic treatment with bumetanide during early development had no effect on body weight or normal behaviors

**Figure 3:**
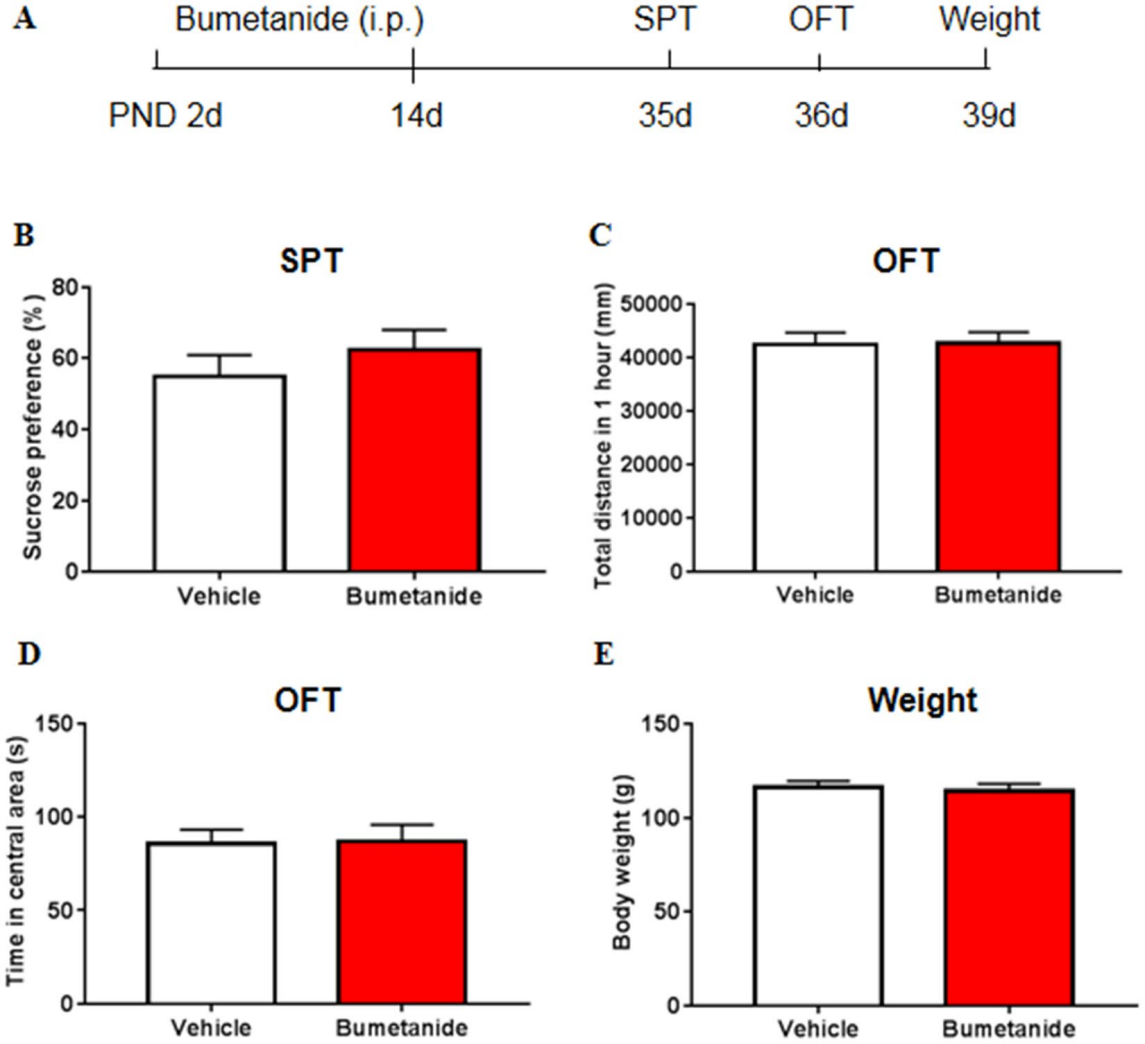
Systemic treatment with bumetanide during early development had no effect on body weight or normal behaviors. (**A**) Experimental timeline of bumetanide treatment and behavioral tests. (**B**) Sucrose preference in the SPT. (**C**) Total distance travelled in 1 h in the open field test (OFT). (**D**) Time spent in the central area in the OFT. (**E**) Body weight of bumetanide and vehicle rats. The data are expressed as mean ± SEM. n = 35–40 per group (13–15 females, 20–23 males).*p < 0.05, compared with vehicle group.

As maternal separation led to increased expression of NKCC1 at P14, and previous studies found bumetanide treatment during a vulnerable developmental period (the first two postnatal weeks) rescues a genetic epilepsy^33^, therefore, we investigated the effects of twice-daily bumetanide treatment during the first two postnatal weeks on body weight, locomotor activity, and emotional behaviors. Bumetanide had no effect on body weight (*t*_73_ = 0.429, *p* = 0.669; Fig. 3E) or locomotor activity, with no difference in the total distance travelled in 1 h (*t*_72_ = 0.066, *p* = 0.948; Fig. 3C). The time in the central area in the open field test (*t*_72_ = 0.116, *p* = 0.908; Fig. 3D) and sucrose preference (*t*_49_ = 0.906, *p* = 0.369; Fig. 3B) were not significantly different between the maternally separated group and control group, indicating that bumetanide did not lead to depressive- or anxiety-like behavior. These data highlight the safety of bumetanide treatment during early development.

### Systemic treatment with bumetanide during early development decreased NKCC1 and GABA_A_ receptor subunit expression after maternal separation

**Figure 4:**
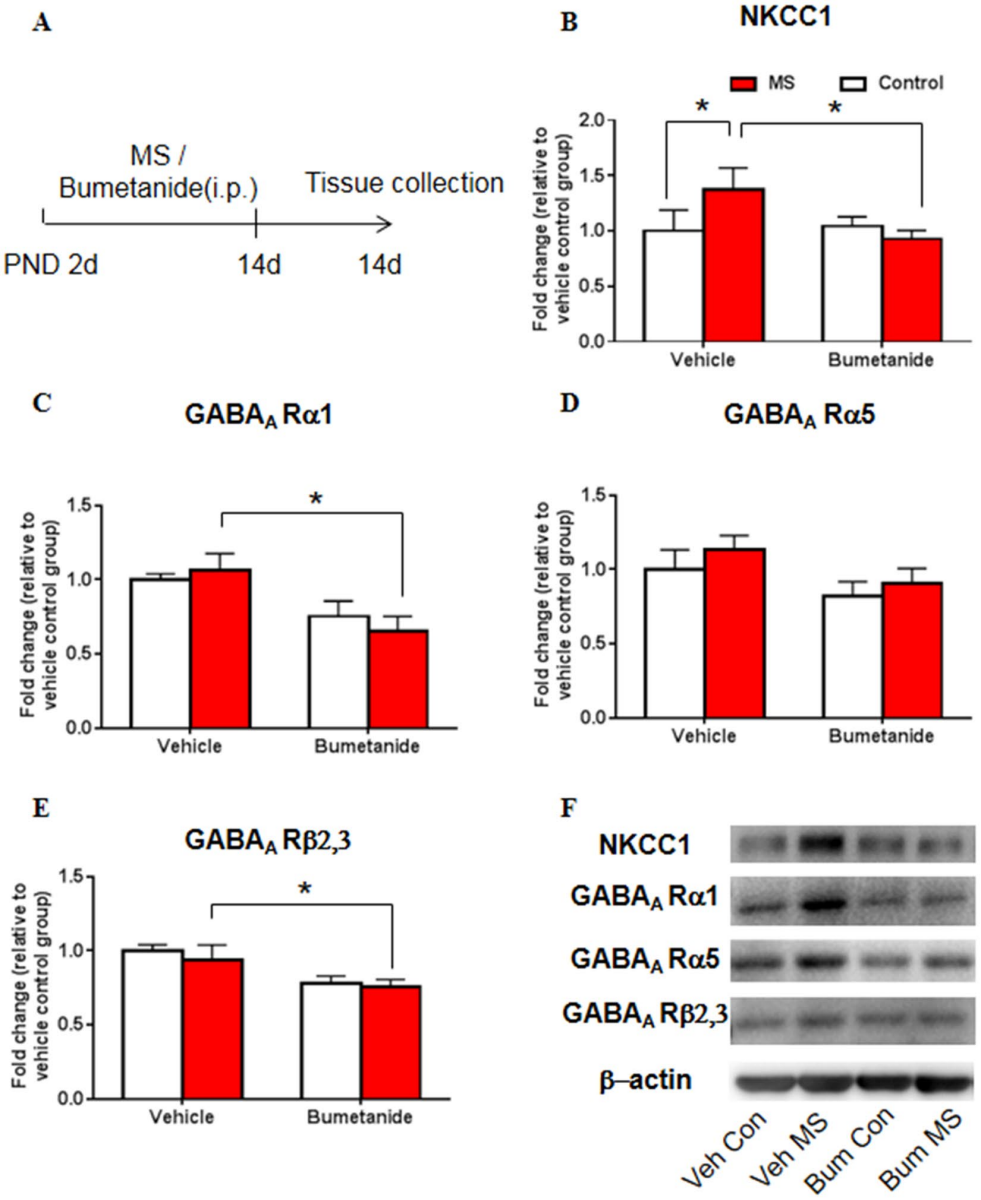
Systemic treatment with bumetanide during early development altered NKCC1 and GABA_A_ receptor subunit expression after maternal separation. (**A**) Experimental timeline of maternal separation, drug treatment, and tissue collection. (**B**–**E**) NKCC1 (**B**), GABA_A_ receptor α1 subunit (**C**), GABA_A_ receptor α5 subunit (**D**), and GABA_A_ receptor β2,3 subunit (**E**) levels in the CA1 area of the hippocampus after maternal separation and bumetanide treatment. (**F**) Representative Western blots in the CA1. Full-length blots are presented in Supplementary Figure S5. n = 10 per group (4 females, 6 males). The data are expressed as mean ± SEM. *p < 0.05.

Then we examined the expression of NKCC1 and GABA_A_ receptor subunits following MS and bumetanide treatment to determine whether bumetanide restores impairments in NKCC1 expression and the GABAergic system that are induced by MS. The two-way ANOVA, with drug treatment (vehicle and bumetanide) and stress (control and MS) as the between-subjects factors, was used to analyze the effects of NKCC1 inhibition on the expression of NKCC1 and GABA_A_ receptor subunits. No significant main effects or interaction were found for the expression of NKCC1 (treatment: *F*_3,19_ = 2.701, *p* = 0.117; stress: *F*_3,19_ = 1.096, *p* = 0.308; treatment × stress: *F*_3,19_ = 3.990, *p* = 0.060; Fig. 4B). A significant main effect of treatment on the expression of the GABA_A_ receptor α1 subunit was found (treatment: *F*_3,19_ = 11.808, *p* = 0.003; stress: *F*_3,19_ = 0.033, *p* = 0.858; treatment × stress: *F*_3,19_ = 0.676, *p* = 0.421; Fig. 4C). A significant effect of treatment on the expression of the GABA_A_ receptor α5 subunit was found (treatment: *F*_3,19_ = 5.230, *p* = 0.034; stress: *F*_3,19_ = 1.539, *p* = 0.230; treatment × stress: *F*_3,19_ = 0.058, *p* = 0.812; Fig. 4D). A significant effect of treatment on the expression of the GABA_A_ receptor β2,3 subunit was also found (treatment: *F*_3,19_ = 8.563, *p* = 0.009; stress: *F*_3,19_ = 0.350, *p* = 0.561; treatment × stress: *F*_3,19_ = 0.073, *p* = 0.791; Fig. 4E). The *post hoc* test showed that MS significantly upregulated the expression of NKCC1 (*p* = 0.049; Fig. 4B), which could be reversed by bumetanide treatment (*p* = 0.016; Fig. 4B). Bumetanide significantly reduced the expression of the GABA_A_ receptor α1 subunit (*p* = 0.006; Fig. 4C) and β2,3 subunit (*p* = 0.040; Fig. 4E) in maternally separated rats compared with the vehicle group. These results suggest that bumetanide restored the MS-induced increase in NKCC1 expression and reduced GABA_A_ receptor subunit expression in the hippocampus.

### Systemic treatment with bumetanide during early development restored behavioral and molecular abnormalities in adolescent rats after maternal separation

Next, we investigated the effects of twice-daily bumetanide treatment on MS-induced depressive- and anxiety-like behavior. After maternal separation or bumetanide treatment from P2 to P14, each rat was subjected to the forced swimming as the stressor. We then conducted the SPT, EPM test, open field test (OFT), and novelty-suppressed feeding test (NSFT) to assess depressive- and anxiety-like behavior that was induced by MS and swim stress during adolescence. After the behavioral tests, the brain tissue were collected for western blotting to examine the expression of NKCC1, KCC2 and GABA_A_ receptor subunits.

**Figure 5:**
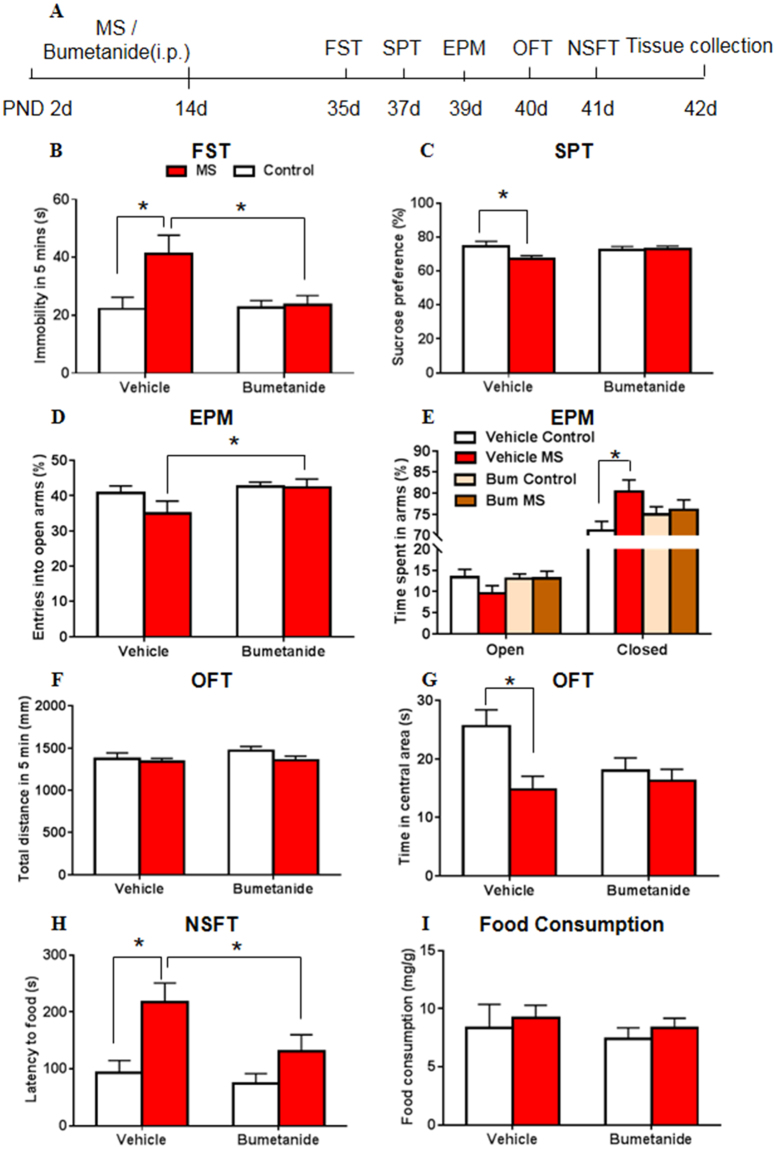
Systemic treatment with bumetanide during early development restored behavioral abnormalities in adolescent rats after maternal separation. (**A**) Experimental timeline of maternal separation, drug treatment, and behavioral tests. (**B**) Immobility time in the FST. (**C**) Sucrose preference in the SPT. (**D**) Percentage of entries into the open arms in the EPM. (**E**) Time spent in arms in the EPM. Time spent in the open arms is plotted as a percentage of the total observation time. (**F**) Total distance travelled in 5 min in the OFT. (**G**) Time spent in the central area in the OFT. (**H**) Latency to feed in the novelty-suppressed feeding test (NSFT). (**I**) Food consumption in the homecage over 5 min in the NSFT. n = 16–20 per group (8–10 females, 8–10 males). The data are expressed as mean ± SEM. *p < 0.05.

The two-way ANOVA, with drug treatment (vehicle and bumetanide) and stress (control and MS) as the between-subjects factors, was used to analyze the effects of NKCC1 inhibition on the behavioral measures. A significant main effect of stress on immobility time was found in the 5 min FST (treatment: *F*_3,73_ = 3.275, *p* = 0.074; stress: *F*_3,73_ = 4.537, *p* = 0.037; treatment × stress: *F*_3,73_ = 3.617, *p* = 0.061; Fig. 5B). In the SPT, the analysis revealed no significant main effects on sucrose preference or interaction (treatment: *F*_3,67_ = 0.673, *p* = 0.415; stress: *F*_3,67_ = 2.635, *p* = 0.109; treatment × stress: *F*_3,67_ = 2.988, *p* = 0.088; Fig. 5C). In the EPM, the analysis revealed no main effects or interaction for the percentage of entries into the open arms (treatment: *F*_3,71_ = 3.081, *p = *0.084; stress: *F*_3,71_ = 1.299, *p* = 0.258; treatment × stress: *F*_3,71_ = 1.101, *p* = 0.298; Fig. 5D), or for the time spent in open arms (treatment: *F*_3,71_ = 0.818, *p* = 0.369; stress: *F*_3,71_ = 1.120, *p* = 0.293; treatment × stress: *F*_3,71_ = 1.265, *p* = 0.264; Fig. 5E), but a significant main effect of stress for the time spent in closed arms (treatment: *F*_3,71_ = 0.009, *p* = 0.924; stress: *F*_3,71_ = 4.271, *p* = 0.042; treatment × stress: *F*_3,71_ = 2.647, *p* = 0.108; Fig. 5E). In the OFT, a significant main effect of stress on the time spent in the central area was found (treatment: *F*_3,71_ = 1.453, *p* = 0.232; stress: *F*_3,71_ = 6.224, *p* = 0.015; treatment × stress: *F*_3,71_ = 3.271, *p* = 0.075; Fig. 5G). No main effects or interaction was found for the total distance travelled (treatment: *F*_3,71_ = 0.988, *p* = 0.324; stress: *F*_3,71_ = 1.581, *p* = 0.213; treatment × stress: *F*_3,71_ = 0.449, *p* = 0.505; Fig. 5F). In the NSFT, a significant main effect of stress on the latency to feed was found (treatment: *F*_3,73_ = 2.809, *p* = 0.098; stress: *F*_3,73_ = 7.992, *p* = 0.006; treatment × stress: *F*_3,73_ = 1.091, *p* = 0.300; Fig. 5H), with no effects or interactions for home cage food consumption (treatment: *F*_3,73_ = 0.580, *p* = 0.449; stress: *F*_3,73_ = 0.645, *p* = 0.425; treatment × stress: *F*_3,73_ = 0.001, *p* = 0.974; Fig. 5I).

The *post hoc* test revealed that MS in the vehicle-treated groups significantly decreased sucrose preference in the SPT (*p* = 0.044; Fig. 5C) and the time spent in the central area in the OFT (*p* = 0.011; Fig. 5G), while increased immobility time in the FST (*p* = 0.016; Fig. 5B), the time spent in closed arms (*p* = 0.025; Fig. 5E) in the EPM and the latency to food in the NSFT (*p* = 0.020; Fig. 5H). Bumetanide reversed the depressive- and anxiety-like behaviors that were induced by MS. Significant differences of immobility time in the FST (*p* = 0.003; Fig. 5B), entries into the open arms in the EPM (*p* = 0.031; Fig. 5D) and latency to food in the NSFT (*p* = 0.046; Fig. 5H) were found between bumetanide MS group and vehicle MS group. The *post hoc* test revealed no differences in total distance travelled (*p* > 0.05; Fig. 5F) in the OFT or home cage food consumption (*p* > 0.05; Fig. 5I) in the NSFT between groups.

**Figure 6:**
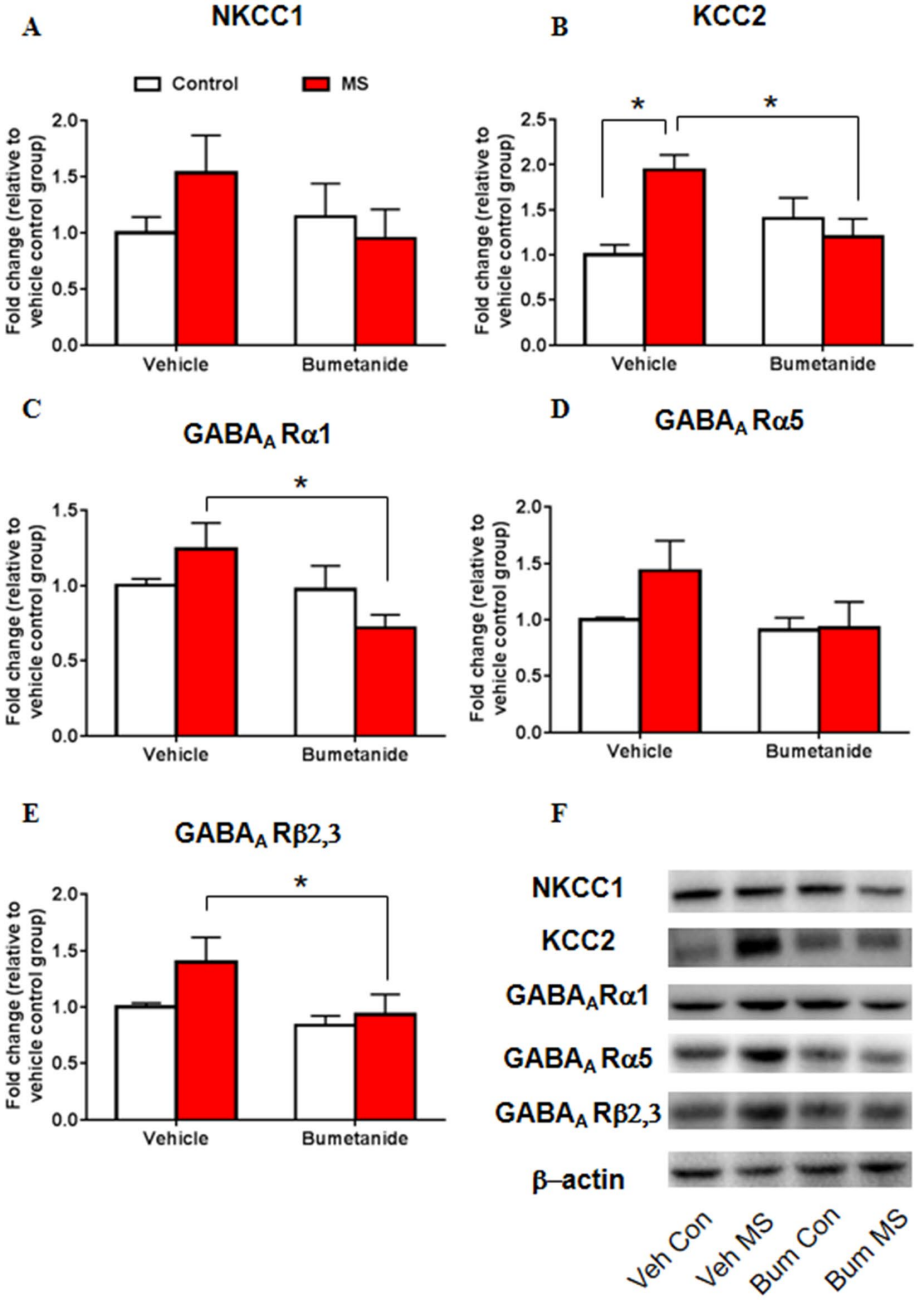
Systemic treatment with bumetanide during early development altered KCC2 and GABA_A_ receptor subunit expression in adolescent rats after maternal separation. (**A**–**E**) NKCC1 (**A**), KCC2 (**B**), GABA_A_ receptor α1 subunit (**C**), GABA_A_ receptor α5 subunit (**D**), and GABA_A_ receptor β2,3 subunit (**E**) levels in the CA1 area of the hippocampus after maternal separation and bumetanide treatment. (**F**) Representative Western blots in the CA1. Full-length blots are presented in Supplementary Figure S6. n = 12 per group (6 females, 6 males). The data are expressed as mean ± SEM. *p < 0.05.

The two-way ANOVA was also used to analyze the effects of NKCC1 inhibition on the expression of NKCC1, KCC2 and GABA_A_ receptor subunits. No significant main effects or interaction were found for the expression of NKCC1 (treatment: *F*_3,16_ = 0.727, *p* = 0.406; stress: *F*_3,16_ = 0.437, *p* = 0.518; treatment × stress: *F*_3,16_ = 2.008, *p* = 0.176; Fig. 6A). Significant main effect of stress and interaction were found for the expression of KCC2 (treatment: *F*_3,16_ = 0.939, *p* = 0.347; stress: *F*_3,16_ = 4.519, *p* = 0.049; treatment × stress: *F*_3,16_ = 10.833, *p* = 0.005; Fig. 6B). A significant main effect of treatment on the expression of the GABA_A_ receptor α1 subunit (treatment: *F*_3,16_ = 4.803, *p* = 0.044; stress: *F*_3,16_ = 0.003, *p* = 0.957; treatment × stress: *F*_3,16_ = 3.940, *p* = 0.065; Fig. 6C) and GABA_A_ receptor β2,3 subunit (treatment: *F*_3,16_ = 4.523, *p* = 0.049; stress: *F*_3,16_ = 2.814, *p* = 0.113; treatment × stress: *F*_3,16_ = 1.035, *p* = 0.324; Fig. 6E) were found. No significant effects or interaction was found on the expression of the GABA_A_ receptor α5 subunit (treatment: *F*_3,16_ = 2.664, *p* = 0.122; stress: *F*_3,16_ = 1.530, *p* = 0.234; treatment × stress: *F*_3,16_ = 1.268, *p* = 0.277; Fig. 6D). The *post hoc* test showed that MS significantly upregulated the expression of KCC2 (*p* = 0.001; Fig. 6B), which could be reversed by bumetanide treatment (*p* = 0.008; Fig. 6B). Bumetanide significantly reduced the expression of the GABA_A_ receptor α1 subunit (*p* = 0.009; Fig. 6C) and β2,3 subunit (*p* = 0.041; Fig. 6E) in maternally separated rats compared with the vehicle group.

These results suggest that bumetanide restored abnormalities in emotional behavior and also the increases in KCC2 expression and GABA_A_ receptor subunit expression in adolescent rats that were exposed to MS and adolescent swim stress.

### Systemic treatment with bumetanide during early development restored behavioral abnormalities in adult rats after maternal separation

Finally, we investigated the effects of twice-daily bumetanide treatment on MS-induced depressive- and anxiety-like behavior in adult rats. After maternal separation or bumetanide treatment from P2 to P14, each rat was subjected to the forced swimming as the stressor at P35. We then conducted the SPT, EPM test, open field test (OFT), and FST to assess depressive- and anxiety-like behavior that was induced by MS and swim stress during adulthood. After the behavioral tests, the serum of rats were collected for osmolality test.

**Figure 7:**
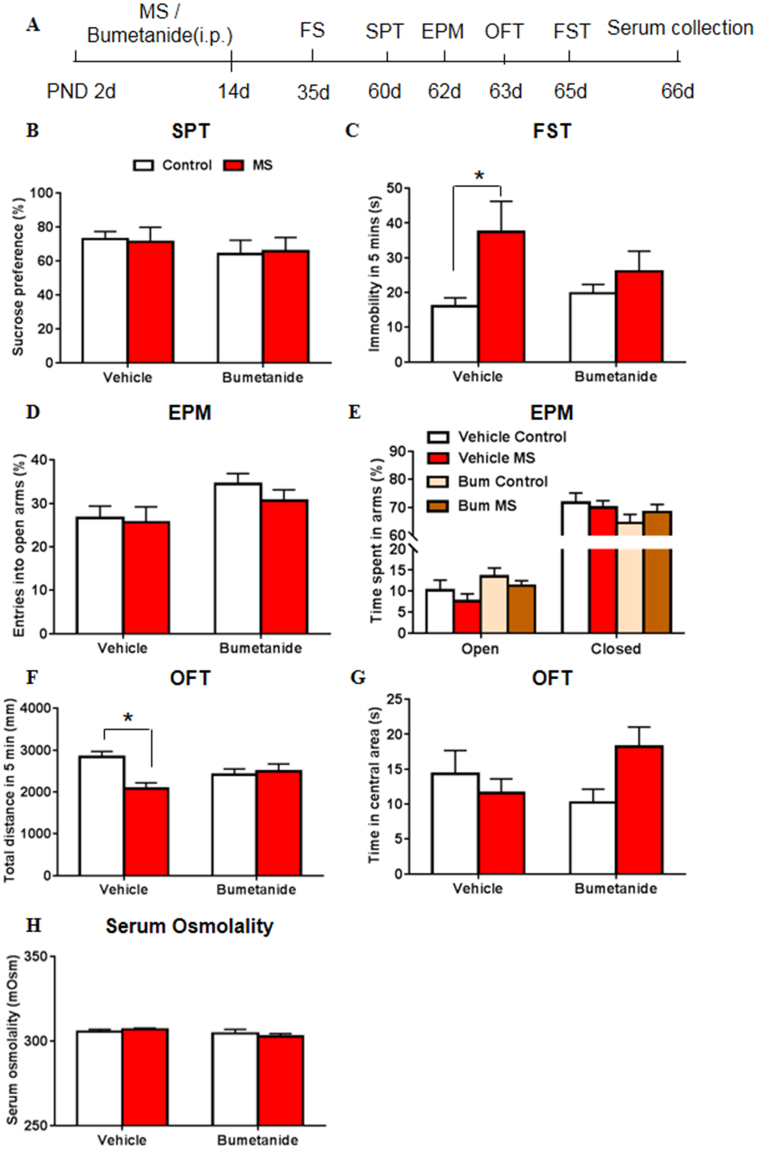
Systemic treatment with bumetanide during early development restored behavioral abnormalities in adult rats after maternal separation. (**A**) Experimental timeline of maternal separation, drug treatment, and forced swimming (FS) stress during adolescence and behavioral tests in adult rats. (**B**) Sucrose preference in the SPT. (**C**) Immobility time in the FST. (**D**) Percentage of entries into the open arms in the EPM. (**E**) Time spent in arms in the EPM. Time spent in the open arms is plotted as a percentage of the total observation time. (**F**) Total distance travelled in 5 min in the OFT. (**G**) Time spent in the central area in the OFT. (**H**) Serum osmolality of each group. The standard curve of osmolality test was shown in Fig S3. n = 12 per group (6 females, 6 males).The data are expressed as mean ± SEM. *p < 0.05.

The two-way ANOVA, with drug treatment (vehicle and bumetanide) and stress (control and MS) as the between-subjects factors, was used. The analysis revealed no significant main effects or interaction for sucrose preference in the SPT (treatment: *F*_3,35_ = 0.876, *p* = 0.356; stress: *F*_3,35_ = 0.001, *p* = 0.990; treatment × stress: *F*_3,35_ = 0.048, *p* = 0.828; Fig. 7B), and for the time in open arms (treatment: *F*_3,39_ = 3.398, *p = *0.073; stress: *F*_3,39_ = 1.580, *p* = 0.216; treatment × stress: *F*_3,39_ = 0.011, *p* = 0.918; Fig. 7E) and closed arms (treatment: *F*_3,39_ = 2.205, *p = *0.146; stress: *F*_3,39_ = 0.136, *p* = 0.715; treatment × stress: *F*_3,39_ = 0.881, *p* = 0.354; Fig. 7E) in the EPM test. A significant main effect of stress on immobility time was found in the FST (treatment: *F*_3,42_ = 0.0.488, *p* = 0.489; stress: *F*_3,42_ = 6.364, *p* = 0.016; treatment × stress: *F*_3,42_ = 1.874, *p* = 0.178; Fig. 7C). In the EPM, the analysis revealed a significant main effect of treatment for the percentage of entries into the open arms (treatment: *F*_3,39_ = 4.894, *p = *0.033; stress: *F*_3,39_ = 0.703, *p* = 0.407; treatment × stress: *F*_3,39_ = 0.249, *p* = 0.620; Fig. 7D). In the OFT, a significant main effect of stress and interaction on the total distance travelled were found (treatment: *F*_3,42_ = 0.004, *p = *0.952; stress: *F*_3,42_ = 5.194, *p* = 0.028; treatment × stress: *F*_3,42_ = 8.171, *p* = 0.007; Fig. 7F), with no significant main effects or interaction for the time spent in the central area (treatment: *F*_3,42_ = 0.007, *p = *0.932; stress: *F*_3,42_ = 0.434, *p* = 0.513; treatment × stress: *F*_3,42_ = 3.082, *p* = 0.086; Fig. 7G). The *post hoc* test revealed that MS in the vehicle-treated groups significantly increased immobility time in the FST (*p* = 0.009; Fig. 7C) and decreased total distance travelled in the OFT (*p* = 0.001; Fig. 7F). Bumetanide reversed the depressive- and anxiety-like behaviors that were induced by MS.

At last, we examined whether the effect of NKCC1 inhibition on behaviors was associated with serum osmolality. The two-way ANOVA, with drug treatment (vehicle and bumetanide) and stress (control and MS) as the between-subjects factors, was used. The analysis revealed no significant main effects or interaction for serum osmolality (treatment: *F*_3,41_ = 2.470, *p* = 0.124; stress: *F*_3,41_ = 0.029, *p* = 0.886; treatment × stress: *F*_3,41_ = 0.894, *p* = 0.350; Fig. 7H). The *post hoc* test also revealed no differences (*p* > 0.05; Fig. 7H) between groups.

These results suggest that bumetanide reversed the depressive- and anxiety-like behaviors in the FST and OFT that were induced by MS in adult rats, and adolescence may be a more vulnerable time to MS-induced stress than adulthood.

## Discussion

In the present study, we found that MS led to depressive- and anxiety-like behavior in adolescent rats and increased the expression of NKCC1, KCC2, and GABA_A_ receptor subunits in the CA1 area of the hippocampus. Intraperitoneal injection of the NKCC1 inhibitor bumetanide during early development did not affect body weight, locomotor activity, or depressive- or anxiety-like behavior in naive rats. However, bumetanide reversed the behavioral abnormalities that were caused by MS and decreased NKCC1, KCC2, GABA_A_ α1 and β2,3 subunit levels in the hippocampus. Moreover, bumetanide did not affect serum osmolality in adult rats. Altogether, our results suggest that NKCC1 in the hippocampus may play an important role in maintaining the function of GABAergic transmission and regulating the vulnerability to MS-induced stress.

Previous studies demonstrated that MS for 180 min per day from P2 to P14 or to P21 induced depression-like behaviors during adolescence^38,39^, and no significant difference was observed in the 15-min-per-day MS group^39^. Consistent with these studies, we found that MS increased the susceptibility to stress in adolescent rats. Differences in the MS protocol between the present study and previous studies include the duration of daily MS and total number of days of MS (i.e., separation for 15 or 180 min per day and from P2 to P14 or to P21), and these differences in MS intensity may lead to different behavioral outcomes. In the present study, we found a variety of behavioral changes observed in behavioral tests. Sucrose preference and immobility time in the FST are core symptoms of depression, which reflect the depression-like state, such as anhedonia and despair^36,40^. The EPM and NSFT are well-established behavioral model of anxiety^40^, and the OFT was used to measure the exploratory locomotor activity of animals^41^. Previous studies reported that maternal separation would not change basal sucrose preference in adolescent rats^42^ and adult rats^43–45^, which were consistent with our findings. Maternal separation reduced sucrose preference in adolescent rats which experienced forced swim stress but not in adult rats, and the differences may suggest adolescence may be a more vulnerable time to MS-induced stress than adulthood^46^. Previous studies also found that MS did not alter the time in the open arms in the EPM^43^ in adult rodents. Additionally, we found that MS increased immobility time in adolescent rats, whereas sucrose preference was unchanged. However, rats that experienced MS and forced swimming during adolescence showed decreased sucrose preference. This indicates that sucrose preference may reflect the depression-like state in rats that did not experience stress during adolescence, but forced swimming itself could act as a kind of stressor to detect stress vulnerability^10,47^. Previous studies also showed that rats experienced MS from P2 to P14 and restraint stress for 2 weeks during adolescence exhibited a decrease in sucrose preference, whereas no change was observed in rats that were not subjected to restraint stress^43,44^. Commons *et al*. recently discussed that the forced swim test measures coping strategy to an acute inescapable stress and thus provides unique insight into the neural limb of the stress response^47^. According to the Two-Hit hypothesis, the etiology of neuropsychiatric disorders usually involves multiple stressors experienced subsequently during different phases of life^7,10^. Based on these findings, we used forced swim stress during adolescence to measure the rats’ response to a second stress later in life^10^.

Growing evidence indicates that the hippocampus and amygdala are involved in ELS-related mental illness, such as depression^15,16^. Brain imaging studies found that the volume of the hippocampus in patients with depression is significantly smaller than normal control groups, whereas the volume of the left amygdala presents a tendency toward a decrease in patients with depression^48^. Both aged and adolescent patients with depression presented a reduction of effective connectivity from the amygdala to the hippocampus^49,50^. Repeated injections of corticosterone, which is an animal model of depression, led to alterations in GABAergic and glutamatergic activity, such as the expression of GAD67, GAD65, and several GABA_A_ receptor subunits in the hippocampus and amygdala^51^. These results suggest that the hippocampus and amygdala play an important role in depression. Furthermore, a meta-analysis found that hippocampal volume was significantly reduced in children who suffered from childhood abuse, with no significant alterations in the volume of the amygdala^52^. These results may suggest that the hippocampus plays a more important role in stress vulnerability that is induced by ELS.

Cation-chloride cotransporters play an important role in neuronal development, including neuronal proliferation, migration, differentiation, and the wiring of neuronal networks through the modulation of GABA receptor-mediated synaptic transmission^19,53^. In the rodent cortex, the expression of KCC2 rapidly increases during development, consistent with intense synaptogenesis^54^. A greater proportion of KCC2 in cortical neurons is found in dendritic spines or their vicinity^55^, indicating that CCCs may be involved in the formation of dendritic spines. Additionally, a previous study found that interfering with the early depolarizing effect of GABA with the NKCC1 inhibitor bumetanide prolonged critical-period plasticity in visual cortical circuits. In the control group, the plasticity of the visual cortex was reduced at the end of the critical period of development (i.e., about 35 days after birth), whereas the visual cortex retained high plasticity at 35 days after birth in the bumetanide intervention group^24^. Therefore, CCCs may play a role in the depolarizing effect of GABA and selectively modulate cortical plasticity.

Derangements of CCCs are involved in various neuropsychiatric disorders, such as epilepsy, neuropathic pain, schizophrenia, autism, and Down’s syndrome^18^. The downregulation of KCC2 and upregulation of NKCC1 were found in the hippocampus in adult patients with temporal lobe epilepsy, leading to the depolarization of GABA_A_ receptor-mediated activity in CA1 pyramidal cells^56^. In rodent models of autism, the GABA excitatory-inhibitory shift is abolished during delivery, leading to greater excitatory GABA transmission and autism-like behavior^25^. Stress can also lead to changes in the expression of CCCs. Chronic unpredictable mild stress led to an increase in NKCC1 expression in the hypothalamic paraventricular nucleus, resulting in the loss of GABA inhibition^57^. Similarly, higher expression of NKCC1 and lower expression of KCC2 in the hippocampus were observed in rats that were stressed by forced water administration using esophageal intubation^58^. Maternal restraint stress also resulted in an increase in the NKCC1/KCC2 ratio in the hippocampus at 14 and 28 days after birth^59^. In the present study, we found that MS increased NKCC1 expression in the CA1 area of the hippocampus on P14, which may be associated with greater susceptibility to stress in adolescent rats.

NKCC1 inhibition by bumetanide has been shown to be effective in the treatment of various neurological and psychiatric disorders^29–31^. Clinical studies have reported a therapeutic effect of bumetanide in temporal lobe epilepsy^60^ but not newborn seizures in a clinical trial^35^. Bumetanide treatment also attenuated seizures in a genetic animal model of epilepsy^33^ and neonatal rats after hypoxia-ischemia^61^. The synaptic plasticity and memory deficits in a mouse model of Down’s syndrome were restored by 1-week treatment with bumetanide^26^. A therapeutic effect of bumetanide was also found in an animal model of autism^25^, which was supported by a case report of childhood autism^62^. Although bumetanide inhibits both NKCC1 and NKCC2, NKCC2 mainly existed in kidney, and bumetanide could cross the brain-blood barrier^63^, we believe it was an optimal choice to selectively block NKCC1 in the brain. On the other hand, it is still unclear about the effects of NKCC1 antagonism on behavioral abnormalities that are induced by stress. In the present study, bumetanide treatment during early development restored the depressive- and anxiety-like behaviors that were induced by MS, which may extend the application of bumetanide to the field of stress vulnerability that is related to ELS.

The precise mechanisms that underlie the regulatory role of CCCs in the vulnerability to stress are unclear. We speculate that CCCs may be associated with GABAergic transmission. Alternations of NKCC1, KCC2, and GABA_A_ receptor subunits have been observed in many brain diseases, such as experimental models of seizure^64,65^ and Fragile X syndrome^27,66^. Lower brain concentrations of GABA and alterations in GABA_A_ receptor subunit expression were found in depressive patients^67^. A negative allosteric modulator of α5 subunit-containing GABA_A_ receptors exerted a rapid and sustained antidepressant-like action in mice^68^. During early development, GABA_A_ receptors that contain α5, β2, β3, and γ subunits are essential for the depolarizing action of GABA^69^. Early life stress may lead to long-term changes in GABA_A_ receptors^70^, such as higher expression of the GABA_A_ α4βδ subunit in the hippocampal CA1 region^71^. Previous studies found that prenatal stress altered GABA_A_ receptor α1 and α5 subunit expression levels^59^. In the present study, MS increased the expression of the GABA_A_ receptor α1 and β2,3 subunits in the hippocampus, and bumetanide treatment decreased the expression of GABA_A_ receptor subunits compared with the vehicle groups. These findings indicate that stress influences the early depolarizing action of GABA, and these impairments can be restored by bumetanide. Additionally, manipulations of NKCC1 during early development led to the prolongation of cortical plasticity and lower density of perineuronal nets^24^, suggesting that the effect of CCCs on MS-induced stress vulnerability may be associated with neuronal plasticity and its regulators, such as brain-derived neurotrophic factor and perineuronal nets.

In summary, the present study shows that NKCC1 in the hippocampus plays an important role in regulating the stress response that is related to ELS. Maternal separation induced depressive- and anxiety-like behaviors in adolescent rats, which could be rescued by bumetanide administration during early development. The present findings suggest a new potential target for the treatment of ELS-related psychiatric disorders.

## Methods

### Animals and housing

Pregnant female Sprague-Dawley rats were purchased from the Center of Laboratory Animal Science, Peking University Health Science Center. The rats were pregnant for 13–15 days at the time of arrival in the laboratory. They were individually housed under a 12 h/12 h light/dark cycle with *ad libitum* access to water and food. All of the animal procedures were performed in accordance with the National Institutes of Health Guide for the Care and Use of Laboratory Animals and were approved by the Biomedical Ethics Committee for Animal Use and Protection of Peking University.

### Maternal separation

The day of delivery was denoted as postnatal day 0 (P0)^72^. The number of pups were limited to 10–12 pups per litter in order to eliminate confounding effects of litter size or differences in maternal care^73^ (5 litters per group). Maternal separation was applied from P2 to P14. Pups were removed from their home cage and isolated from their mothers in other cages for 3 h (9:00 AM–12:00 PM) during the dark period. For separation during the first week, the isolation boxes were placed in a humidified incubator at 32 °C. Control rats were reared together with their dam without disturbance^74^. For each experimental group, pups were separated from their mother at weaning age (P21). All the pups of one group was temporarily put into one cage, and then randomly divided into cages each containing up to five of the same gender per cage. The data presented in bars is comprised by male and female rat pooled data according to previous studies^44,75,76^.

### Pharmacological treatment

From P2 to P14, the pups were injected i.p. twice daily with 0.2 mg/kg bumetanide (Sigma) or vehicle (2% dimethylsulfoxide in physiological saline) according to previous studies^24,26,33,63^. The final concentration of the drug is 0.1 mg/ml. Therefore, in our study the volume was less than 0.05 ml per injection using gauge 26 beveled needles during the first postnatal week. Apart from regular injection instructions, the needle was stayed for a while in case of drug leak. Bumetanide or vehicle was diluted in physiological saline immediately before the injection.

### Serum collection and osmolarity measurement

Rats were decollated and blood was collected immediately. Samples were centrifuged and osmolarity of recovered serum was estimated by a Vapor pressure osmometer^24^.

### Behavioral tests

#### Forced swim test

The forced swim test (FST) is a well-established behavioral model of despair^37^. Forced swimming during adolescence (P35) served as a second stressor^47^ and was also used to test for depressive-like behavior^10^. The FST was conducted during the dark period under dim light. Plastic cylinders (50 cm height, 24 cm diameter) contained tap water (25 °C ± 1 °C) to a depth of 35 cm. The animals were unable to touch the cylinder’s bottom with their hind paws or tail. The FST consisted of two sessions on two consecutive days (15 min pretest and 5 min test). The rats were individually placed in the cylinders for 15 min on the first day and then placed again in the cylinders 24 h later. The total duration of immobility (immobility time) was measured during a 5 min test. The behavior of each animal was simultaneously monitored by a video camera. Immobility was defined as the absence of any active movements other than those necessary to keep the head and nose above the water (e.g., when the rats floated in a vertical position). After each session, the rats were removed from the water, dried with a towel, placed in a warm enclosure, and then returned to their home cage. The videos were analyzed using EthoVision XT 10. The EthoVision XT 10 analyzed the behavior video based on manually set mobile and immobile thresholds. To find the correct threshold we follow the suggestion from engineer of EthoVision XT, and then we randomly chose two animals in each experiment and recorded the immobility time by two independent observers who were blind to the animal groups, and finely adjusted the immobile threshold based on the recorded time in each experiment.

#### Open field test

The open field test (OFT) was performed to assess exploratory locomotor activity in an unfamiliar environment as a rat model of depression, and this model mimics some aspects of human depression, including lower levels of activity, and lack of interest^41^. The test was conducted in a dark room. The apparatus consisted of a gray box that was open on top. The dimensions of the box were 100 cm × 100 cm × 40 cm. The floor was divided into 25 equal squares (20 cm × 20 cm). The arena was illuminated with a 40 W lamp in the center, 60 cm above the floor. Each rat was gently placed in the center square of the floor of the arena, and behavior was videotaped for 5 min or 1 h for locomotor activity assessment. After each rat was tested, the box was thoroughly cleaned to remove odor cues. Locomotor activity and the time spent in the central area were analyzed using EthoVision XT 10. We detected animal dwelling time in central area with the body of the rat was in the zone.

#### Sucrose preference test

The sucrose preference test (SPT) is considered an index of anhedonia^77^. The SPT was conducted during the dark period under dim red light according to previous studies^40,41^. The rats were habituated to a 1% sucrose solution for 48 h before the test day, during which water and 1% sucrose were offered in two identical bottles. The locations of these two bottles were interchanged every 24 h. The rats were deprived of food and water for 4 h, followed by a 1 h preference test, in which water and 1% sucrose were offered in two identical bottles. During the test, the locations of the bottles were interchanged every 30 min. The water and sucrose bottles were weighed to determine sucrose preference.

#### Novelty-suppressed feeding test

The novelty-suppressed feeding test (NSFT) was performed according to previously established protocols^78^. A more ‘anxious’ animal will take more time to begin eating in a novel environment^79^. The rats were deprived of food for 24 h and then placed in the corner of an open field arena (75 cm × 75 cm × 40 cm), and a pellet of food was placed on a white piece of paper (10 cm × 10 cm) in the center of the cage. Each test lasted 10 min, and the latency to approach the food and begin eating was recorded as the main test parameter. The latency to feed was recorded when the rat sat on the paper square and bit the pellet using its forepaws. Subsequent home cage food consumption over 5 min was the quantitative control measure for appetite.

#### Elevated plus maze

The EPM is based on a rat’s natural fear of open, unprotected, and elevated spaces and is considered an index of anxiety^40^. The EPM consisted of four arms (two open arms and two closed arms, each 50 cm long and 10 cm wide) that were arranged in a plus configuration and elevated 70 cm above the floor. The closed arms had 40 cm high walls. Illumination was 3 lux in the closed arms and 8 lux in the open arms. Each rat was first placed in the central zone of the EPM with its head facing an open arm. The rat was allowed to freely explore the maze for 5 min. The percentage of entries into the open arms and time spent on the open and closed arms were recorded from videos^32,80^ and analyzed using EthoVision XT 10. Previous studies counted all four paws into an arm as an entry^81,82^. Therefore, we detected animal dwelling time and entries into arms with the center point of rat was in the arms.

### Western blot assays

The protocols for sample preparation and Western blot were based on our previous studies^83–86^. Bilateral tissue punches (12-gauge) were collected from the CA1 area of the hippocampus and basolateral amygdala. Each group was then divided into 5 or 6 samples based on the number of animals and each sample contained tissues from 2 rats^44^. And we did Western blotting on all samples of each group for every protein listed. And the brain tissues were then homogenized with RIPA lysis buffer (Applygen Technology, Beijing, China) with enzyme inhibitor cocktail (Applygen Technology, Beijing, China). The lysates were centrifuged at 12,000 × *g* for 15 min. The centrifugate was collected, and protein concentrations in all of the samples were quantified using the BCA assay kit (Applygen Technology, Beijing, China). Τhe samples were diluted with RIPA lysis buffer to equalize the protein concentrations. Equal amounts of samples (20 mg) underwent 10% sodium dodecyl sulfate-polyacrylamide gel electrophoresis. The primary antibodies were the following: anti-NKCC1 (1:1000; Cell Signaling Technology, catalog no. 14581 S), anti-KCC2 (1:1000; Abcam, catalog no. ab49917), anti-GABA_A_ receptor α1 subunit (1:1000; Abcam, catalog no. ab33299), anti-GABA_A_ receptor α5 subunit (1:1000; Abcam, catalog no. ab10098), anti-GABA_A_ receptor β2,3 subunit (1:1000; Millipore, catalog no. MAB341), and anti-β-actin (1:1000; Sigma Aldrich, catalog no. A5316). Horseradish peroxidase-conjugated secondary antibody was used (1:2000; anti-rabbit and anti-mouse immunoglobulin G; Santa Cruz Biotechnology and Vector Laboratories, respectively). The levels of all proteins were normalized to the level of β-actin. Band intensities were quantified using Quantity One 4.0.3 (Bio-Rad, Hercules, CA, USA).

### Statistical analysis

The data are expressed as mean±SEM. The statistical analysis of the behavioral and molecular data was performed using Student’s *t*-test and two-way ANOVA followed by Tukey’s *post hoc* test. Values of *p* < 0.05 were considered statistically significant.

## Electronic supplementary material

Supplementary Figure 1–6
