# Supplementary material for: Bumetanide treatment during early development rescues maternal separation-induced susceptibility to stress
Source: Sci Rep. 2017 Sep 19;7:11878. doi: 10.1038/s41598-017-12183-z (PMC5605528; doi:10.1038/s41598-017-12183-z)
Supplement: Supplementary file 1 — Supplementary Figure 1–6 [file 41598_2017_12183_MOESM1_ESM.doc]

August 18, 2017, for *Scientific Reports*

Supplementary information for

**Bumetanide treatment during early development rescues maternal separation-induced** **susceptibility to stress**

**Die Hu,^1, 2^ Zhou-Long Yu,^1, 2^ Yan Zhang,^1, 2^ Ying Han,^1^ Wen Zhang,^1^ Lu Lin,^1, 3, 4^**

**Jie Shi^1, 5, 6^, ***

^1^National Institute on Drug Dependence and Beijing Key Laboratory of Drug Dependence, Peking University, Beijing 100191, China
^2^Department of Pharmacology, School of Basic Medical Science, Peking University Health Science Center, Beijing 100191, China
^3^Peking University Sixth Hospital Peking University Institute of Mental Health, National Clinical Research Center for Mental Disorders, Key Laboratory of Mental Health, Ministry of Health, Peking University, Beijing 100191, China
^4^Peking-Tsinghua Center for Life Sciences and PKU-IDG/McGovern Institute for Brain Research, Beijing 100191, China

^5^State Key Laboratory of Natural and Biomimetic Drugs, Beijing 100191, China

^6^Key Laboratory for Neuroscience of the Ministry of Education and Ministry of Public Healthy, Beijing 100191, China

Abstract: 188 words

Introduction: 631 words

Discussion: 1566 words

Figures: 7

Supplementary information: 1

*Corresponding author:

Prof. Jie Shi

National Institute on Drug Dependence and Beijing Key Laboratory of Drug Dependence, Peking University, 38 Xueyuan Road, Haidian District, Beijing 100191, China

Fax: +86-10-62032624

E-mail: [shijie@bjmu.edu.cn](mailto:shijie@bjmu.edu.cn)

**
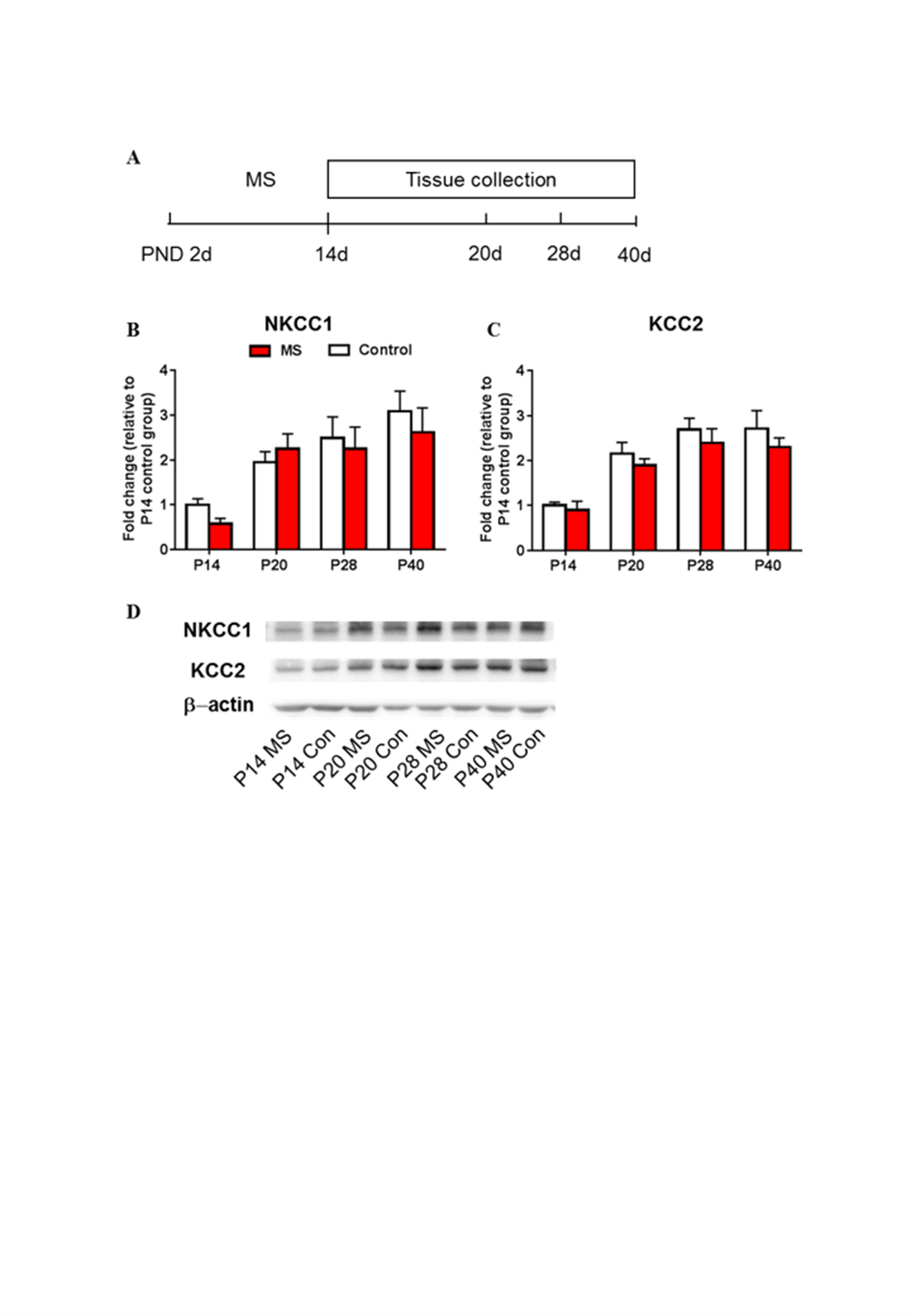
**

**Figure S1. Maternal separation did not alter the expression of NKCC1 or KCC2 in the basolateral amygdala.** (A) Experimental timeline of maternal separation and tissue collection. (B, C) NKCC1 (B) and KCC2 (C) levels in the basolateral amygdala (BLA) on postnatal days 14, 20, 28, and 40. (D) Representative Western blots in the BLA. The data are expressed as mean ± SEM. n = 10 per group (4 females, 6 males). **p* < 0.05, compared with control group at corresponding ages.


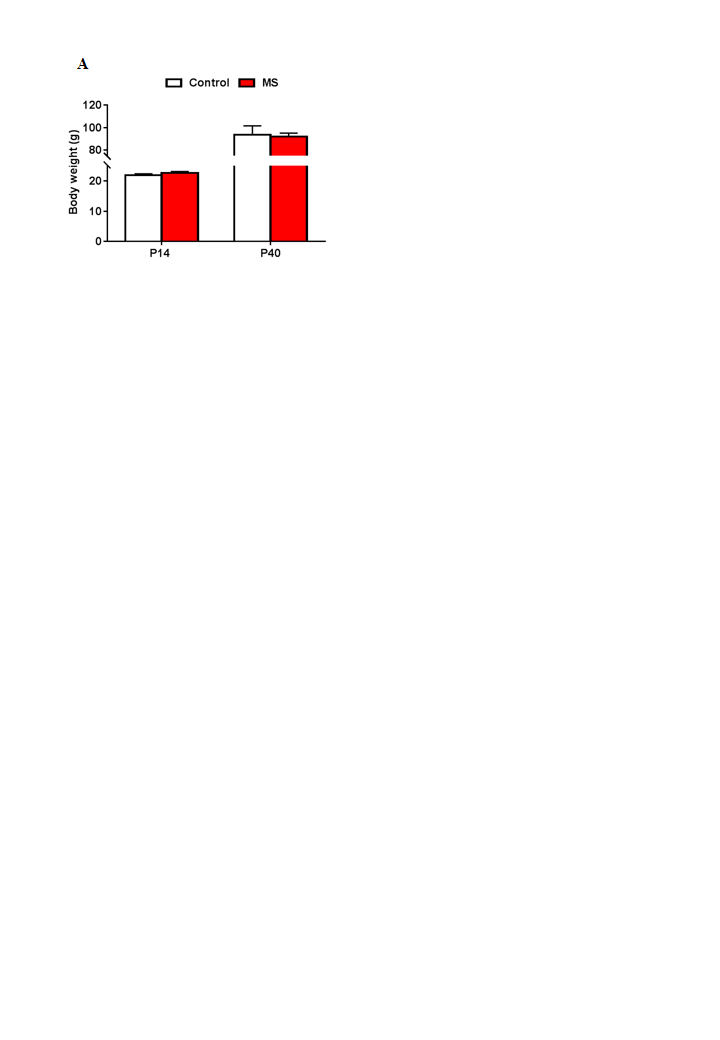


**Figure S2. Body weight of rats**. (A) Body weight of rats of P14 and P40 that underwent maternal separation. n = 16 - 17 per group (7-8 females, 8-10 males).


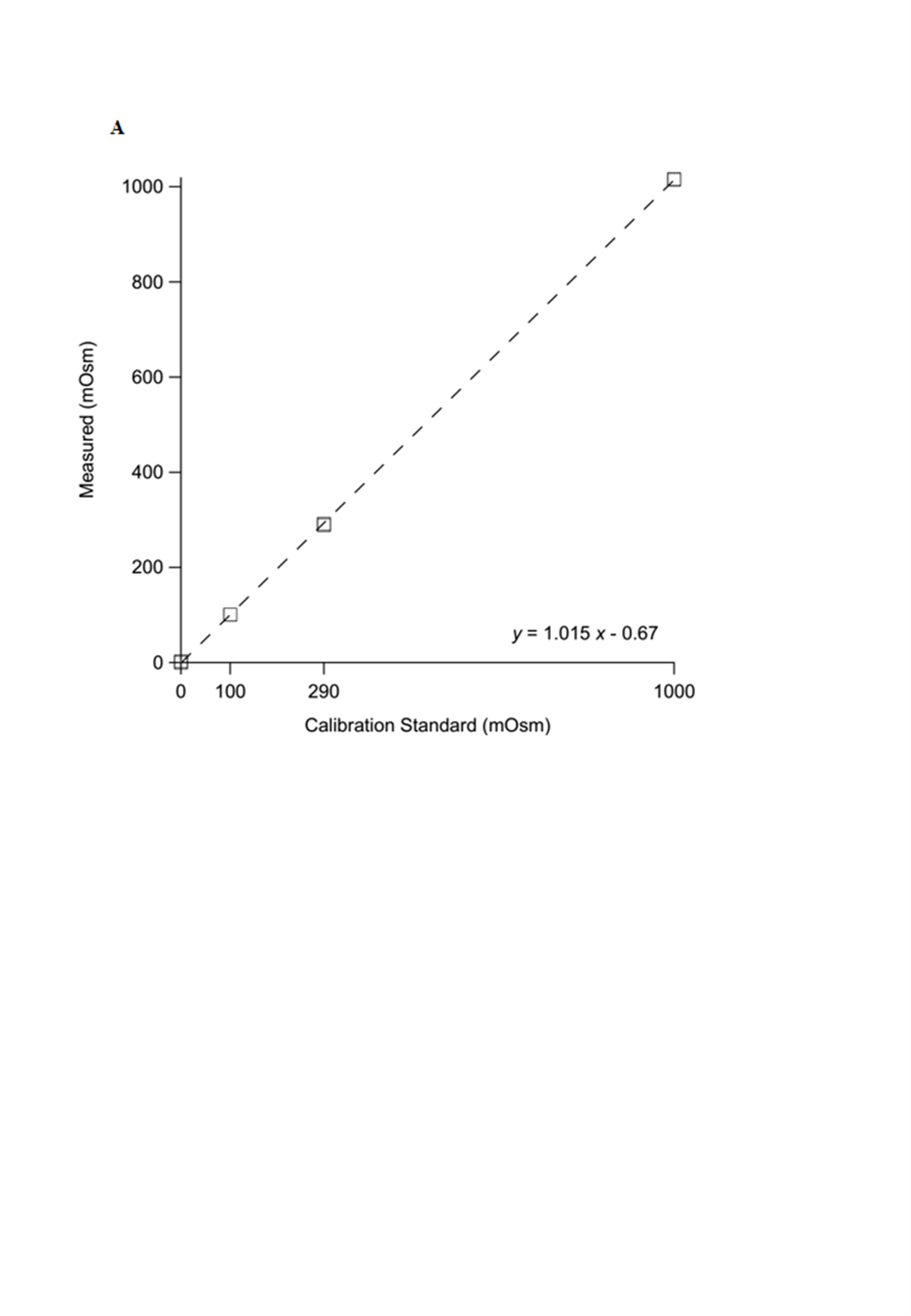


**Figure S3. Standard curve of osmolality test**. (A) Measured osmotic value of standard solutions. The osmolality of standard solutions are 0, 100, 290, 1000. They were repeatedly tested twice and plotted as squares. The values were fitted with a linear equation and result equation was shown on the figure.


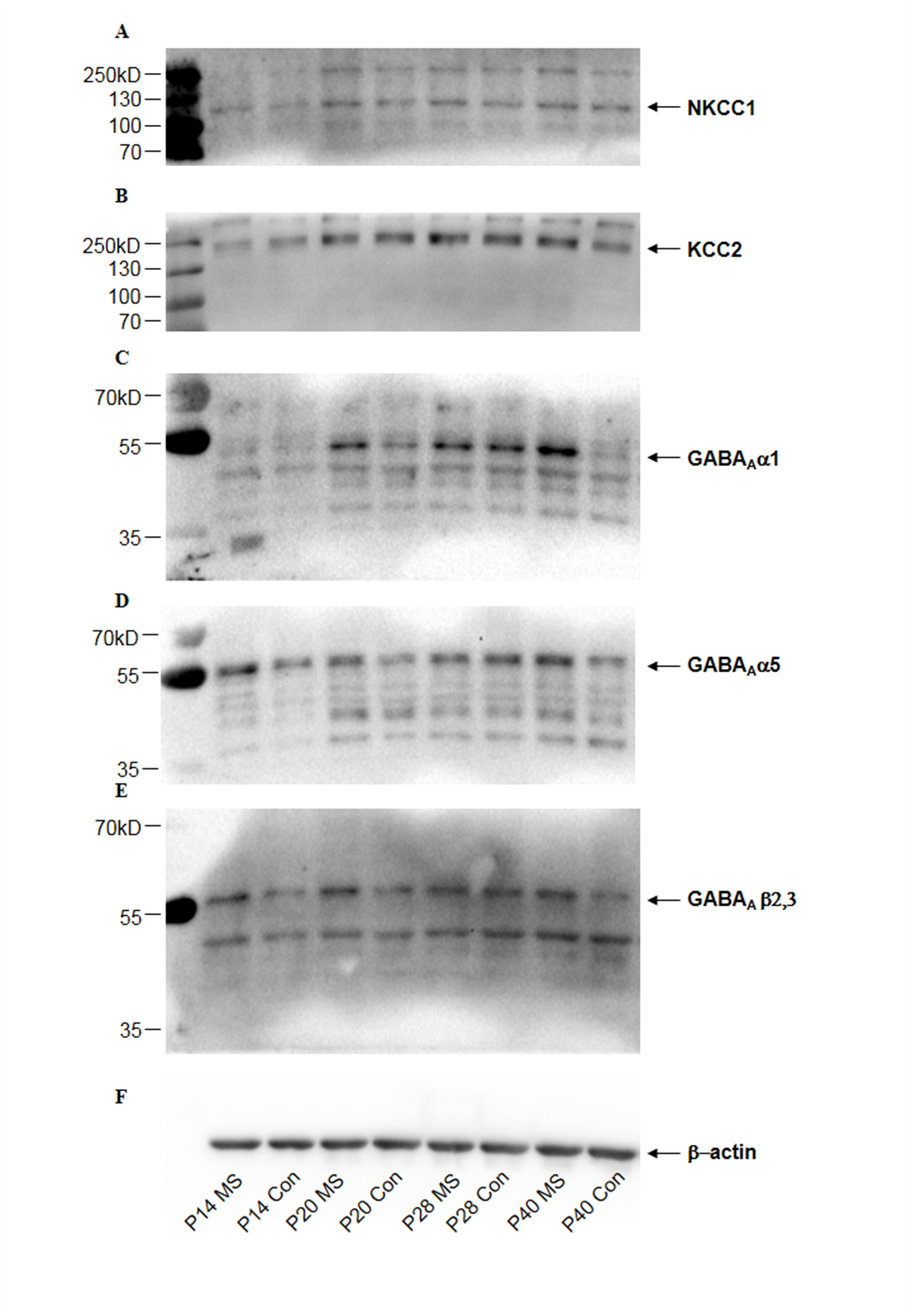


**Figure S4. Full-length blots of NKCC1, KCC2, and GABA_A_ receptor subunits in Figure 2**. (A-F) Representative Western blots of NKCC1 (A), KCC2 (B), GABA_A_ receptor α1 subunit (C), GABA_A_ receptor α5 subunit (D), and GABA_A_ receptor β2,3 subunit (E) and β-actin (F) in CA1.


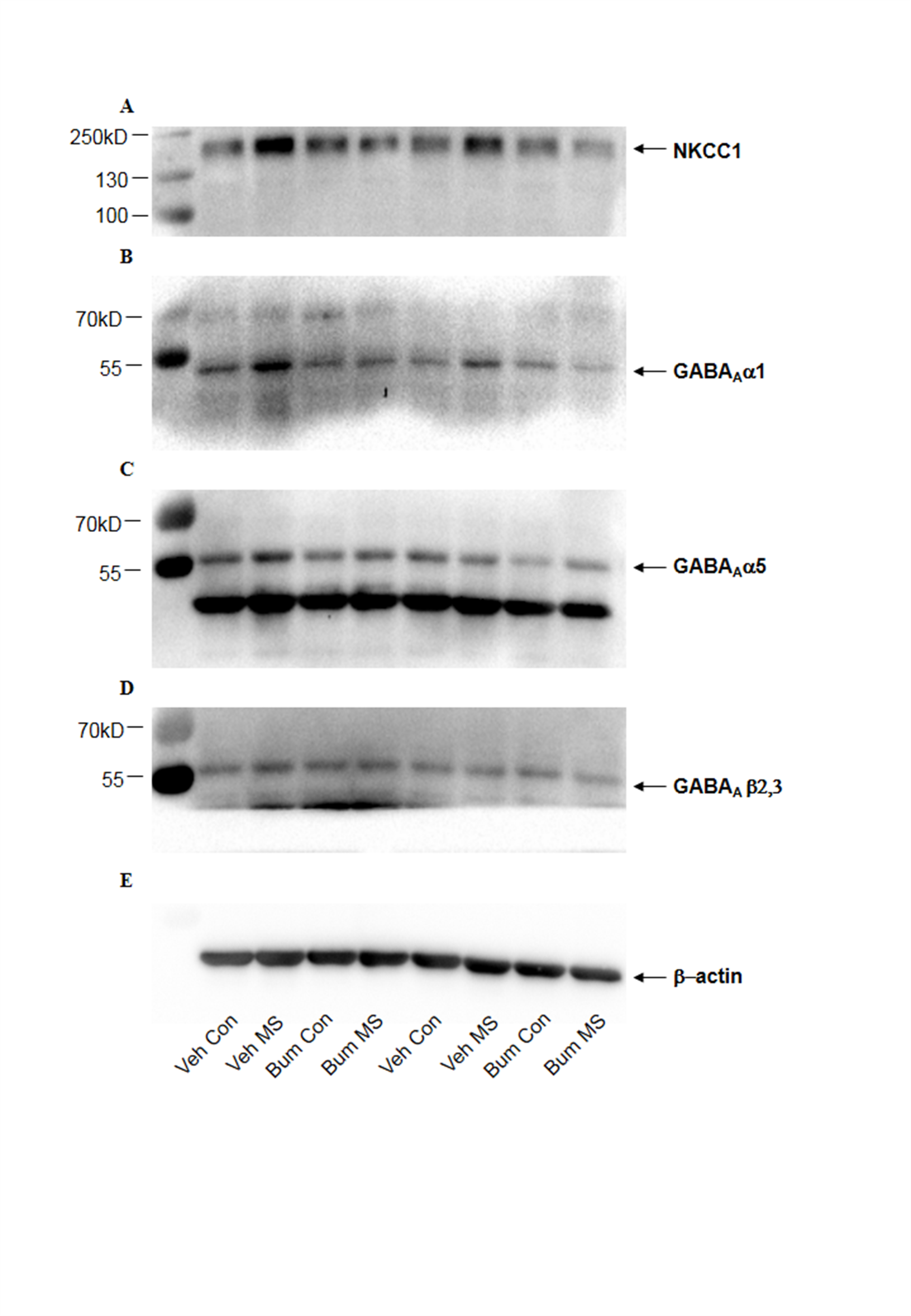


**Figure S5. Full-length blots of NKCC1 and GABA_A_ receptor subunits in Figure 4**. (A-F) Representative Western blots of NKCC1 (A), GABA_A_ receptor α1 subunit (B), GABA_A_ receptor α5 subunit (C), and GABA_A_ receptor β2,3 subunit (D) and β-actin (E) in the CA1.


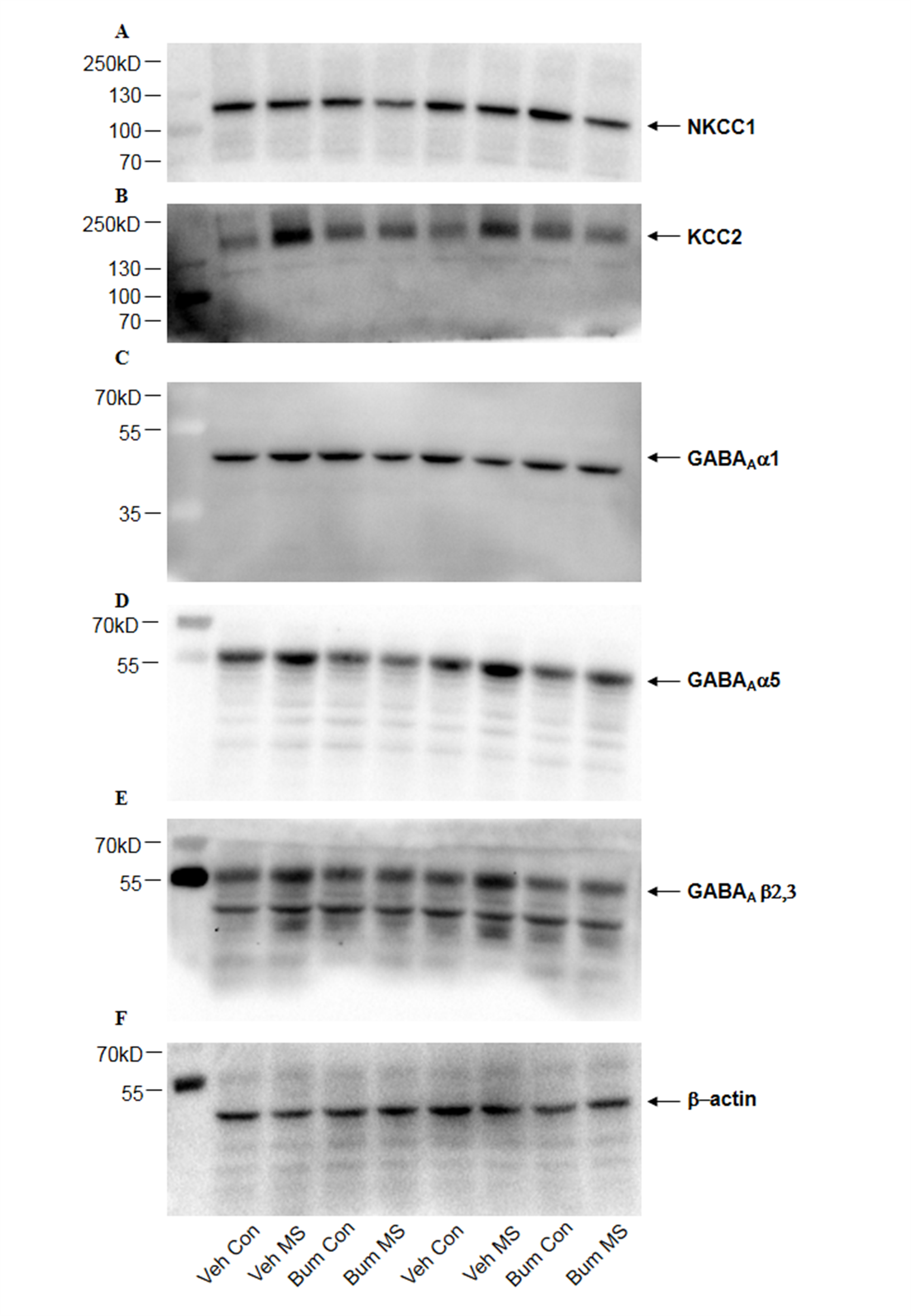


**Figure S6. Full-length blots of NKCC1, KCC2, and GABA_A_ receptor subunits in Figure 6**. (A-F) Representative Western blots of NKCC1 (A), KCC2 (B), GABA_A_ receptor α1 subunit (C), GABA_A_ receptor α5 subunit (D), and GABA_A_ receptor β2,3 subunit (E) and β-actin (F) in the CA1.
